# Hedgehog-Interacting Protein (HIP) Regulates Apoptosis Evasion and Angiogenic Function of Late Endothelial Progenitor Cells

**DOI:** 10.1038/s41598-017-12571-5

**Published:** 2017-09-29

**Authors:** Bom Nae Rin Lee, Yeon Sung Son, Dabin Lee, Young-Jin Choi, Sang-Mo Kwon, Hyun-Kyung Chang, Pyung-Hwan Kim, Je-Yoel Cho

**Affiliations:** 1 Department of Biochemistry, BK21 Plus and Research Institute for Veterinary Science, School of Veterinary Medicine, Seoul National University, Seoul, 151-742 Korea; 2 Laboratory for Vascular Medicine & Stem Cell Biology, Medical Research Institute, Department of Physiology, School of Medicine, Pusan National University, Yangsan, 626-870 Korea; 3 Department of Biomedical Laboratory Science, College of Medical Science, Konyang University, Daejeon, 35-365 Korea

## Abstract

Late endothelial progenitor cells (LEPCs) are derived from mononuclear cells (MNCs) and are thought to directly incorporate into blood vessels and differentiate into mature endothelial cells (ECs). Using transcriptome and proteome analysis, we identified distinctive LEPC profiles and found that Hedgehog-interacting protein (HIP) is strongly expressed in LEPCs. Inhibition of HIP by lentiviral knockdown activated canonical hedgehog signaling in LEPCs, while it activated non-canonical hedgehog signaling in ECs. In LEPCs, HIP knockdown induced much enhanced tube formation and resistance to apoptosis under oxidative stress conditions via canonical hedgehog signaling. Although HIP is strongly expressed in proliferating LEPCs, HIP expression is down-regulated during angiogenesis and under oxidative stress condition. Moreover, when LEPCs are treated with angiogenic triggers such as VEGF and FGF2, HIP expression is reduced. Our findings suggest that HIP blocks LEPC angiogenesis and regulate survival when there is no angiogenic stimulation. HIP inhibition in LEPCs enhanced tube formation and reduced apoptosis, resulting in improved angiogenesis.

## Introduction

Endothelial progenitor cells (EPCs) are circulating blood cells that are capable of promoting vascular repair^1^. EPCs are derived from blood mononuclear cells (MNCs)^2^ and play an essential role in coordinated postnatal vasculogenesis. Following injury to the vasculature of a tissue, hemostasis is initiated, and it provides signals for the mobilization and homing of circulating EPCs. After migrating to the damaged area, EPCs divide, secrete cytokines that support angiogenesis and become incorporated into the vascular network to promote endothelial remodeling and neovasculogenesis^1–3^. In the presence of a stimulus such as a tumor, EPCs also play major roles in promoting tumor vasculature and supporting tumor growth^4,5^.

EPCs are a heterogeneous population of which two subtypes have been identified – earlyEPCs (eEPCs) and lateEPCs (LEPCs). Various groups have reported that LEPCs are a proliferative subtype that form tube-like structures and are directly incorporated into the vasculature. In contrast, eEPCs do not differentiate into mature endothelial cells (EC) and promote angiogenesis indirectly via paracrine mechanisms^6,7^. Based on these physiological differences, only LEPCs are thought to give rise to mature endothelial cells following their differentiation from mononuclear cells (MNCs). Although the contributions of EPCs to ischemia induction or tumor angiogenesis are under intensive investigation, detailed molecular analysis of differentiation focusing on LEPCs is lacking.

Hedgehog (Hh) signaling has gained attention as a key player in postnatal neovasculogenesis^8,9^. In adult heart and skeletal muscle, sonic hedgehog (Shh) has been suggested to directly promote neovascularization and induce secretion of pro-angiogenic growth factors. In tumor angiogenesis, Hh inhibition reduced tumoral VEGF secretion and reduced tumor vasculature and thereby became known for anti-tumor effects^10,11^. Moreover, Hh ligands augment bone marrow-derived eEPC proliferation, migration and VEGF production via Gli-1 dependent canonical Hh signaling^12,13^. However, in mature ECs, Hh proteins increase angiogenesis and migration through RhoA-dependent non-canonical signaling pathways^14,15^. Emerging evidence suggests that Hh signaling has central role in homeostasis and repair processes by tightly regulating angiogenesis.

In this study, using high-throughput RNA sequencing and mass spectrometry-based proteome analysis, we present a comprehensive approach for the characterization of endothelial lineage cells. This study is the first report of using both transcriptomic and proteomic approaches to clarify and characterize LEPCs in the context of physiological differentiation stages. Unbiased expression profiling reveals, for the first time, that Hedgehog-Interacting Protein (HIP) is strongly expressed by LEPCs. HIP is a membrane glycoprotein that inhibits Hh signaling. HIP is known to bind all three Hh ligands (Shh, Ihh and Dhh) with an affinity equal to the affinity of Ptc-1, which is the receptor for Hh ligands^16^. HIP is highly expressed in endothelial cells but down-regulated during angiogenesis and in several types of tumors^17^. In addition, HIP is not present in eEPCs^13^. Therefore, we hypothesized that HIP plays an important role in the tight regulation of LEPC functions.

## Results

### Human LEPCs have distinctive transcriptomic and proteomic profiles

**Figure 1:**
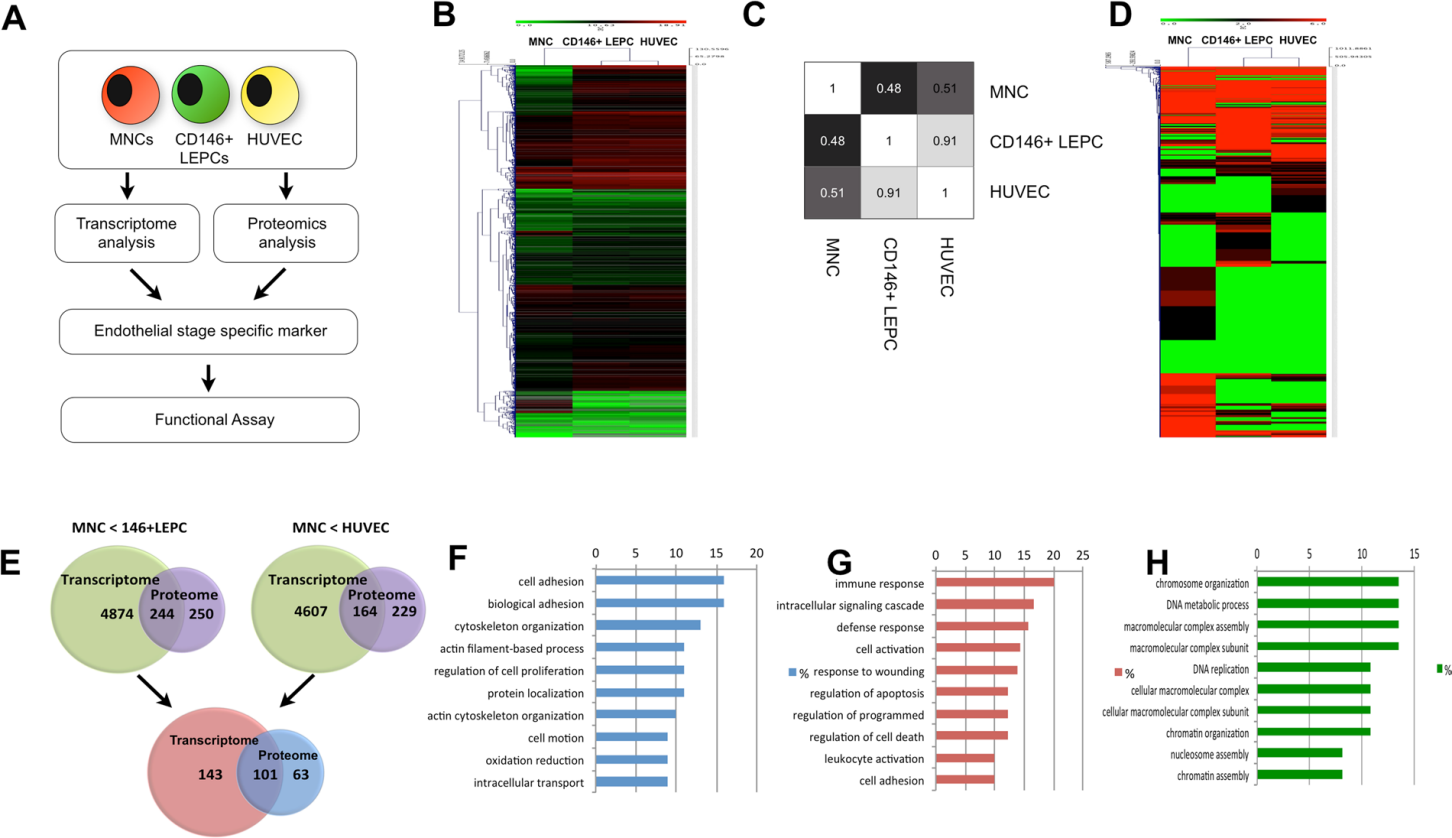
Transcriptome and proteome analysis of MNCs, CD146-positive LEPCs and HUVECs. (**A**) Experimental design used to identify selective molecular signatures for endothelial lineages. (**B**) Heat map demonstrating differential gene expression. The figure was created using Mev software. Red and green colors indicate up-regulated and down-regulated genes, respectively. Genes were further grouped using hierarchical clustering (the distance metric was Euclidean distance, and the linkage method was average). (**C**) Pearson correlation test for the four cell types, MNCs, CD146- + LEPCs and HUVECs. (**D**) Heat map demonstrating differential protein expression. (**E**) The 244 genes indicated in the overlap are those up-regulated at the RNA and protein level in CD146 + LEPCs compared to MNCs. The 164 genes indicated in the overlap on the right were up-regulated in HUVECs compared to MNCS. The 101 genes overlapped in the lower diagram are endothelial signature genes that are up-regulated in both CD146 + LEPCs and HUVECs compared to MNCs. (**F**) The top 10 overrepresented biological processes of endothelial signature genes in GO analysis. (**G**) The top 10 overrepresented biological processes of MNC signature genes in GO analysis. MNC signature genes are genes that are up-regulated in MNCs compared to both LEPCs and HUVECs. (**H**) The top 10 overrepresented biological processes of CD146 + LEPC signature genes compared to HUVECs.

MNCs were isolated from human umbilical cord blood as previously described^18^. LEPCs were obtained by long-term culture (14–21days) of MNCs. We then further isolated CD146-positive LEPCs to further purify the LEPC population by magnetic-activated cell sorting (MACS). Mature vascular endothelial cells (HUVECs) were separated from the human umbilical vein of the same donors from whom we collected cord blood. To investigate the molecular basis of the stage-specific differences, the four progenitor/mature cell populations were used as sources for both high-throughput RNA sequencing (RNA-seq) and semi-quantitative proteomics analysis (Fig. 1A)

Robust and reproducible data were collected, with more than 42 million readings per population. In total, transcripts corresponding to 23,363 genes were identified (Fig. 1B). By performing proteome analysis using LC-MS/MS, we identified a total of 2,127 proteins from all four samples (Fig. 1D). A Pearson correlation analysis using genes differentially expressed among the four populations is consistent with the predicted differentiation hierarchy (Fig. 1C). Principal component analysis revealed that MNCs are a relatively heterogeneous cell type, while CD146-positive LEPCs, HUVECs are more mature and homogeneous cell types. Our data indicate that CD146-positive LEPCs and HUVECs share approximately 91% of their gene expression profiles.

Next, to systematically analyze the transcriptome and proteome at the transitions from MNCs to CD146-positive LEPCs and then to HUVECs, we correlated the transcriptome data with proteome data. We found that 1,947 proteins out of the 2,127 proteins from the proteome data showed expression correlated to the transcriptome data. We identified101 genes as common to LEPCs and HUVECs (Fig. 1F). The 101 genes are listed on the Supplementary Table 1. These genes could serve as endothelial lineage signature genes involved in the endothelial commitment process.

To gain insight into the commitment process, we performed gene ontology (GO) analysis. Gene ontology (GO) analysis indicated that genes up-regulated in MNCs are mainly associated with the immune response and with cell activation, including T cell, leukocyte and lymphocyte activation (Fig. 1G). The 101 genes up-regulated in endothelial commitment mainly participated in cell adhesion, cytoskeletal organization and cell motility (Fig. 1F). This is partly due to the nature of endothelial commitment and differentiation from the circulating blood MNCs because endothelial commitment requires coordinated multistep processes including mobilization, adhesion, transmigration and incorporation. Genes that were uniquely enriched in CD146-positive LEPCs and not in HUVECs participated in chromosome organization, DNA replication and chromatin assembly (Fig. 1H).

### HIP is Selectively Expressed in LEPCs and ECs and is Down-regulated during Angiogenesis and Oxidative Stress

**Figure 2:**
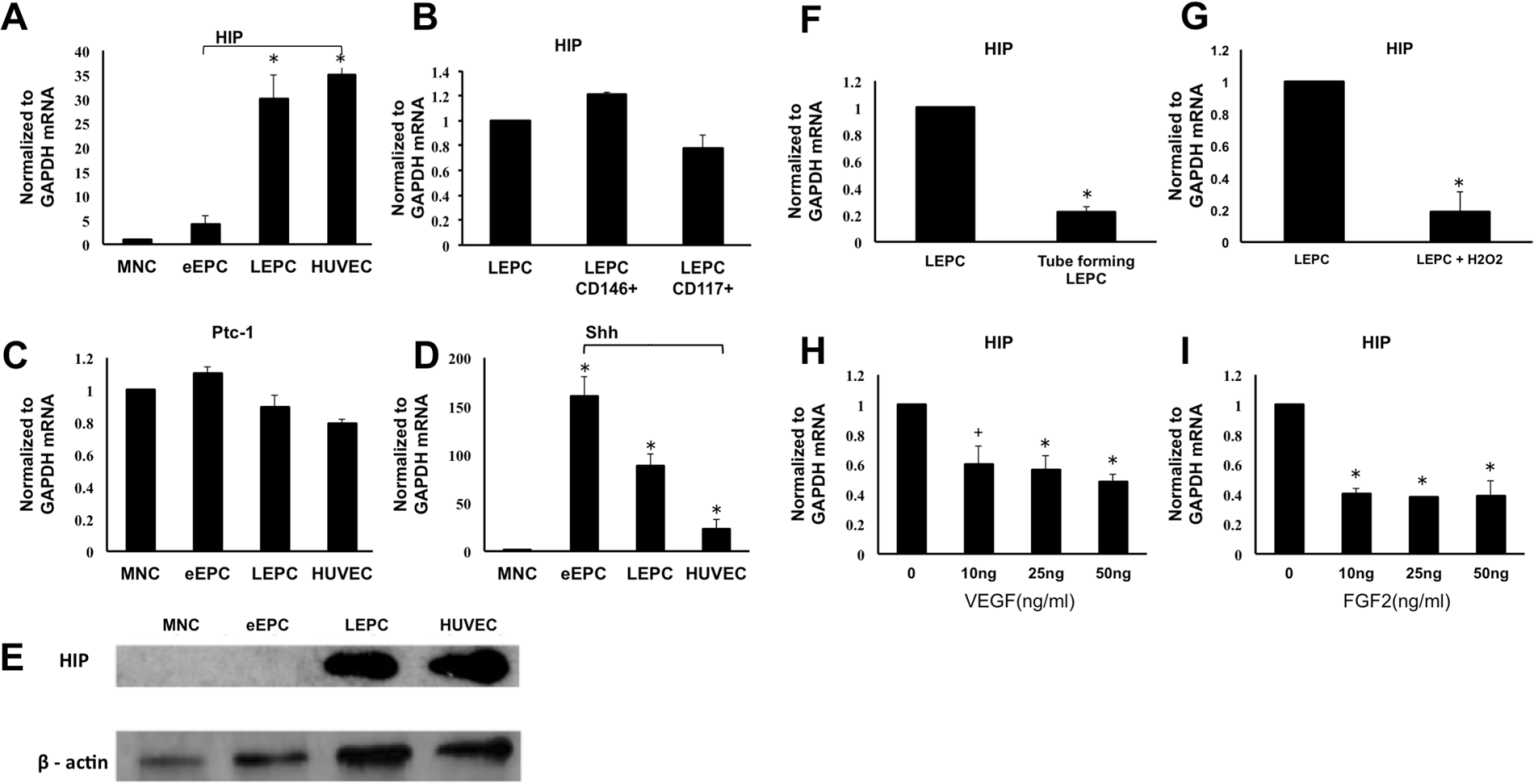
Hedgehog-interacting protein is selectively expressed in late endothelial progenitor cells. Human mononuclear cells and early endothelial progenitor cells were isolated and cultured on fibronectin for 7 days. (**A**) Expression levels of HIP in MNCs, eEPCs, LEPCs and HUVECs assessed by qRT-PCR. (**B**) Expression levels of HIP in LEPCs, CD146 + LEPCs and CD117 + LEPCs. (**C**,**D**) Expression levels of hedgehog target genes (Ptc-1 and Shh) (**E**) Expression level of HIP protein in MNCs, eEPCs, LEPCs and HUVECs. (**F**) Expression levels of HIP in LEPCs and tube forming LEPCs on Matrigel. (**G**) Expression level of HIP in LEPCs and LEPCs under 100 uM H_2_O_2_ (**H**,**I**) LEPCs were treated with FGF2 or VEGF for 16 h, and Hip mRNA expression was measured by qRT-PCR.

Among the 101 genes enriched in the endothelial lineage compared to MNCs, we focused on HIP because of the importance of Hh signaling during developmental angiogenesis. HIP transcripts were confirmed by quantitative real-time RT-PCR and Western blot. In MNCs and eEPCs, HIP expression was very low at the transcript level and was not detected at the protein level (Fig. 2A,E). As both the Hh antagonist HIP and the Hh receptor Ptc-1 are transcriptional targets of Hh signaling, we examined the expression levels of Sonic hedgehog (Shh) and Ptc-1. Interestingly, Ptc-1 expression was similar in MNCs, eEPCs, LEPCs and HUVECs, whereas HIP expression showed a negative correlation with Shh expression among eEPCs, LEPCs and HUVECs (Fig. 2C,D).

Previously, it was shown that HIP is down-regulated during active angiogenesis in HUVEC. We tested the expression of HIP during tube formation, and, indeed, the expression of HIP mRNA extracted from the cells of tubes formed in Matrigel was 64.3% lower than in non-tube forming cells. Moreover, we examined HIP expression under oxidative stress. HIP expression was reduced by more than 30% during treatment with 100 mM H_2_O_2_. Further, the expression of the canonical Hh target Gli-1 was up-regulated in the tube-forming cells (Fig. 2F) and under oxidative stress (Fig. 2G), the cells with lower expression of HIP. Given that it is well established that various cytokines actively promote angiogenesis and tube formation, it is conceivable that HIP is down-regulated by growth factors that promote angiogenesis. Thus, we measured HIP mRNA after treatment of LEPCs with angiogenic growth factors. We observed that VEGF and FGF2 significantly down-regulated HIP mRNA expression (Fig. 2H,I), but this down-regulation was more pronounced after FGF2 treatment than after treatment with the same concentration of VEGF.

### HIP Knockdown Enhanced LEPC Angiogenesis and Mouse Aortic Sprouting

**Figure 3:**
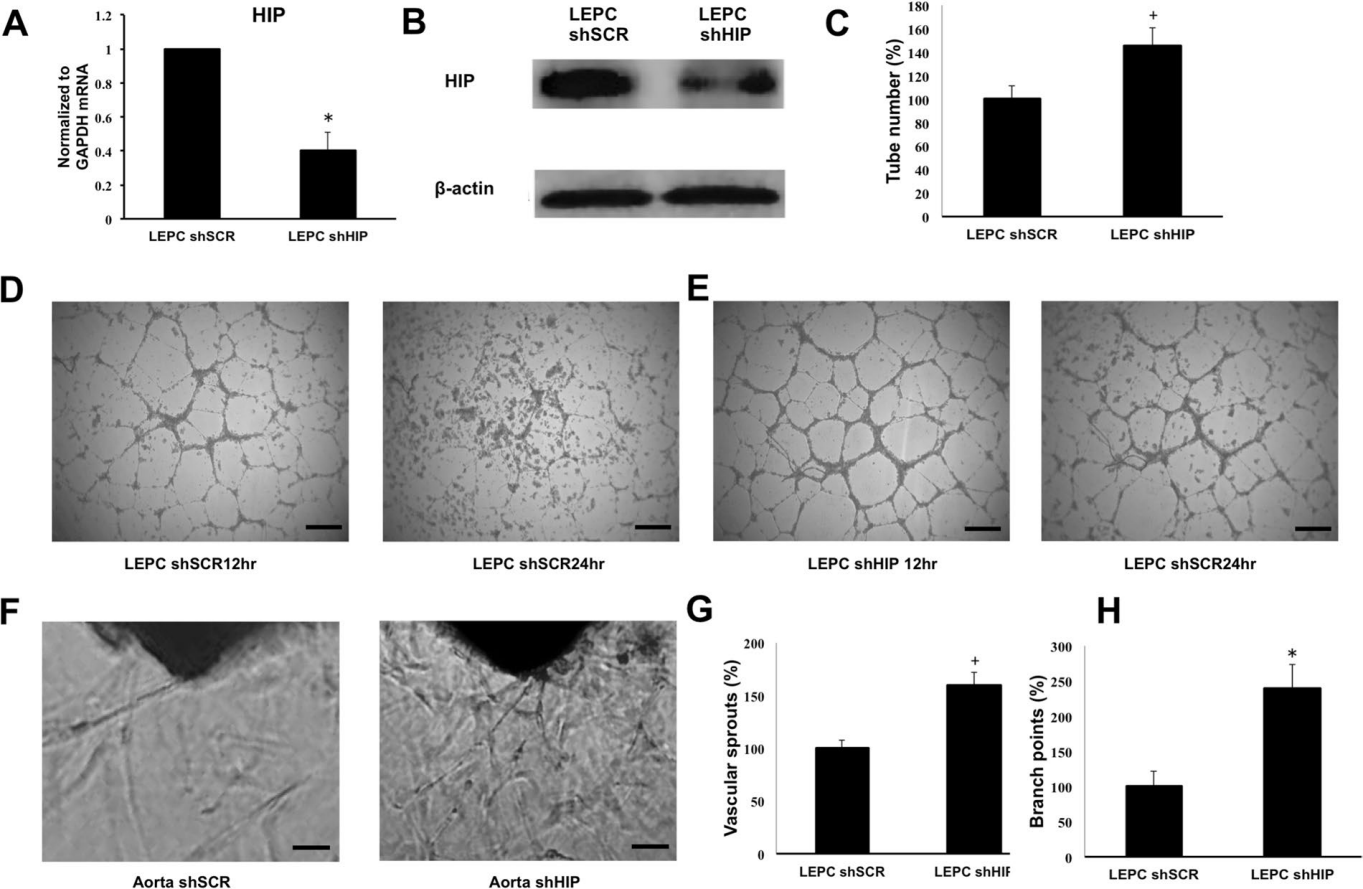
Inhibition of HIP in LEPCs enhances angiogenesis. (**A**,**B**) LEPCs were treated with lentiviral shHIP knockdown vector or scramble vector for 12 h; after 48 h, the downregulation of HIP was confirmed by qRT-PCR and Western blot. (**C–E**) LEPCs were suspended and cultured on Matrigel with 10 ng/ml VEGF. At 12 h and 24 h later, tube formation was assessed under light microscopy, and the tubes were counted. (scale bar, 200 μm) (**F–H**) The aortas of C57BL/6 mice were transduced shHIP lentivirus or scramble containing virus and embedded in 150 μl of Matrigel in EGM2 media with 20 ng/ml VEGF in 96-well plates. After 7 days, micrographs of representative rings were taken, and the total numbers of vascular sprouts and branch points were calculated (scale bar, 100 μm). The values shown are means ± SE (n = 4).

To identify the role of HIP in LEPCs, we generated lentivirus-based HIP shRNA (shHIP). HIP knockdown was confirmed in RNA and protein level (Fig. 3A,B). The effects of HIP knockdown were investigated using the capillary morphogenesis assay. Capillary morphogenesis indicated that HIP knockdown increased the number of tubes formed in Matrigel (Fig. 3C). We found that HIP-inhibited LEPCs formed more durable tubes that lasted longer (Fig. 3D,E). Next, to investigate the functional significance of HIP inhibition *in vivo*, we assessed the aortic sprouting capacity in mice. The thoracic artery was dissected out, and transfected with shHIP lentivirus, and seeded on Matrigel. Figure 3F demonstrates that HIP knockdown enhanced aortic sprouting. The total numbers of vascular sprouts and branching points were increased by more than 50% compared with aortas transfected with scrambled lentivirus (Fig. 3G,H). These findings clearly indicate the functional relevance of HIP knockdown for enhanced LEPC angiogenesis and newly sprouting aorta.

### HIP Knockdown Decreased Apoptosis In LEPCs under Oxidative Stress

**Figure 4:**
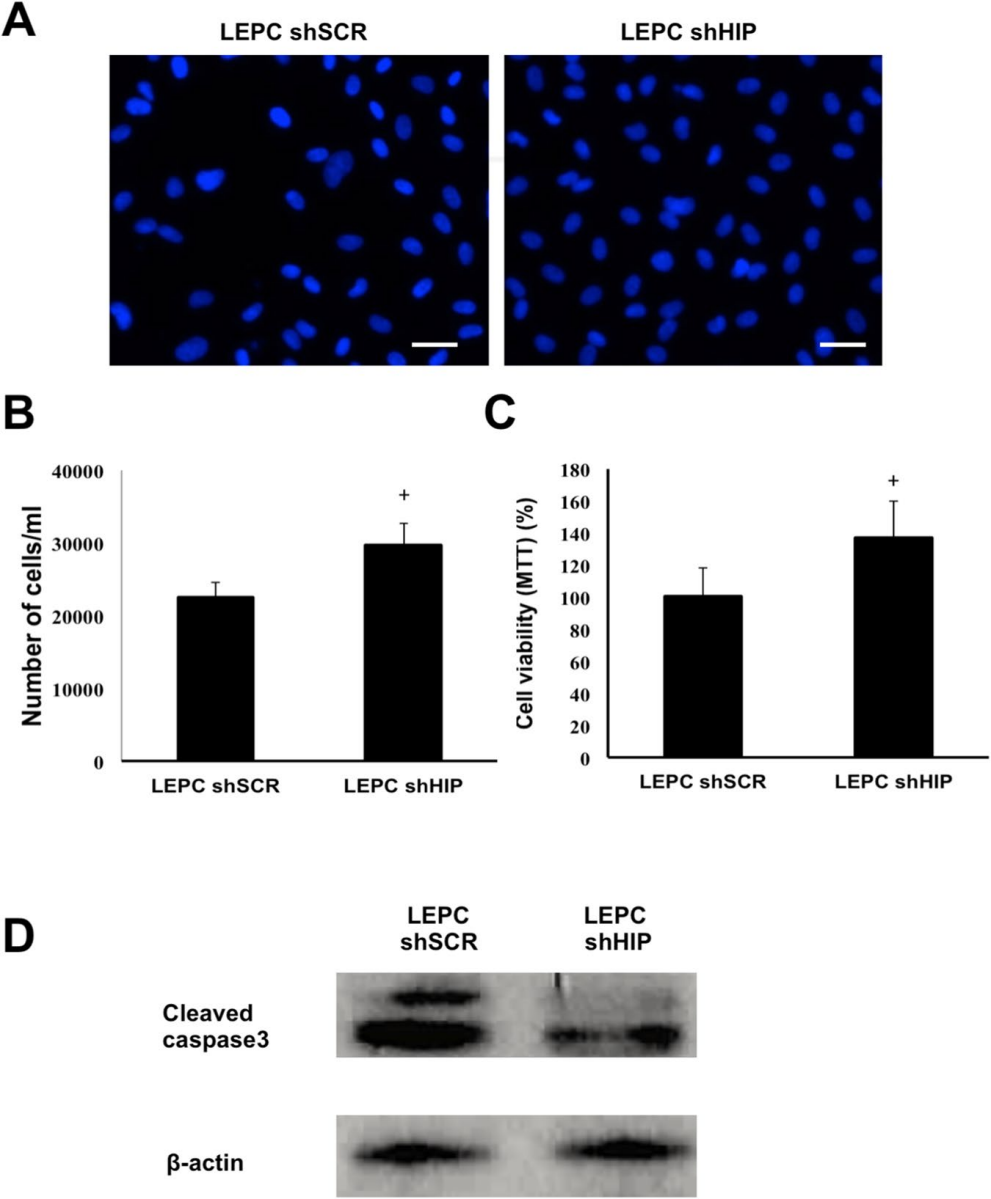
Inhibition of HIP decreases apoptosis under oxidative stress. (**A–C**) LEPCs were treated with 100 mM H_2_O_2_ for 3 h, and dead cells were washed out with PBS. The viable cells were counted and visualized with DAPI staining. (scale bar, 20 μm) Cell viability was assessed using the MTT assay. (**D**) After H_2_O_2_-induced oxidative injury in LEPCs, the level of cleaved caspase-3 was measured by Western blot.

Because LEPCs are faced with oxygen deprivation in ischemic sites where LEPCs are actively involved in angiogenesis, we hypothesized that HIP inhibition would increase resistance to apoptosis and produce decreased proteolytic activation of caspase-3. After LEPCs were treated with 100 mM H_2_O_2_ for 3 hours, cells were washed out and incubated overnight, and then viable cells were detected via DAPI staining and the MTT assay (Fig. 4A–C). LEPCs with low HIP expression were less susceptible to oxidative stress than were normal LEPCs upon treatment with H_2_O_2_. Moreover, LEPCs with low HIP expression showed lower caspase-3 cleavage activity than normal LEPCs after the H_2_O_2_ treatment (Fig. 4D). In the absence of oxidative stress, we measured LEPC proliferation. Interestingly, inhibition of HIP did not affect the proliferation of LEPCs or their expression of the cell cycle regulators cyclin D and E (Supplementary Fig. 2). Next, we hypothesized that HIP knockdown affected LEPC migration and invasion because these are important characteristics of LEPCs in ischemic injury. However, migration and wound closure were not changed by HIP inhibition.

### Hedgehog Protein Activates Canonical Hh Signaling In LEPCs

**Figure 5:**
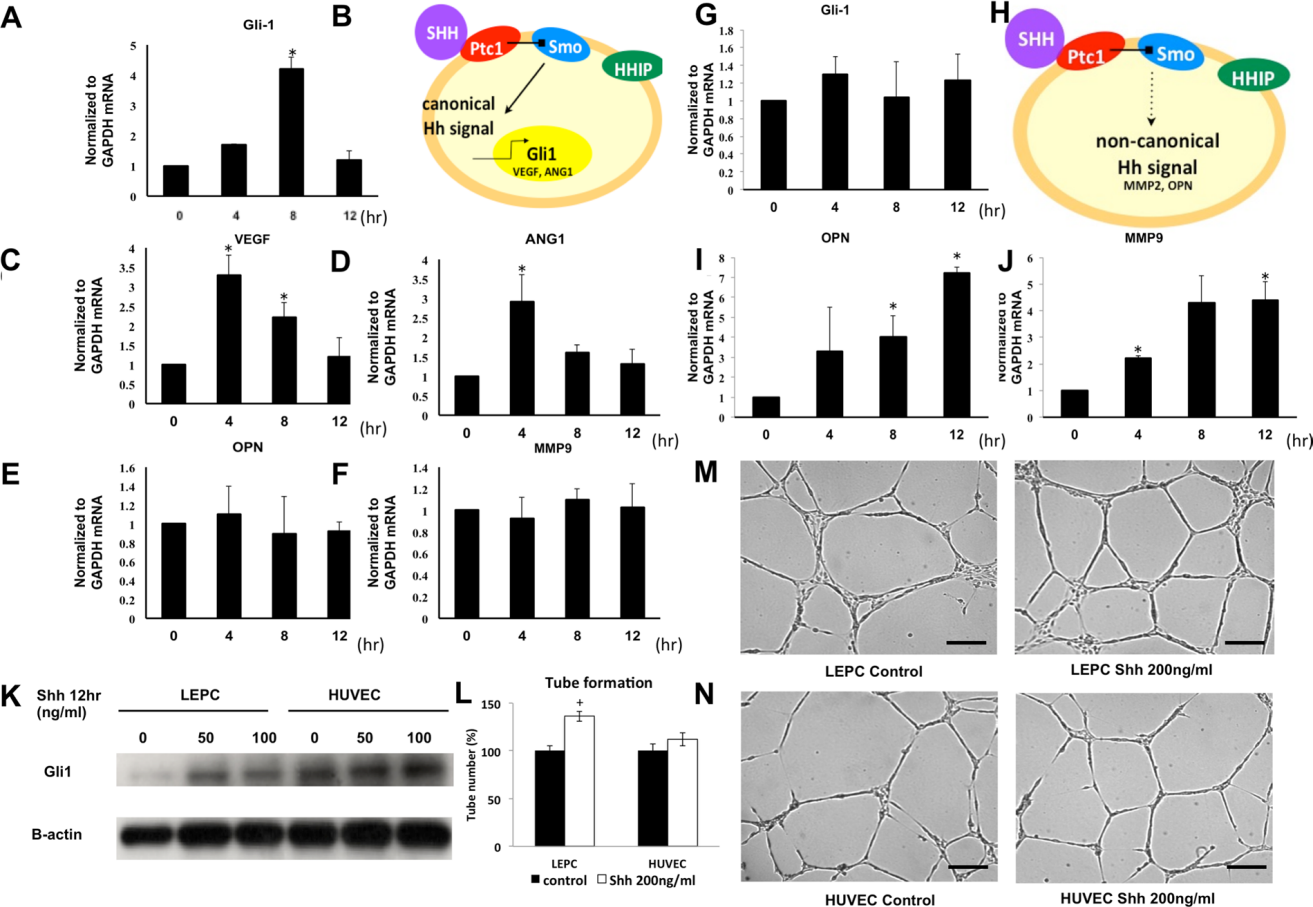
Shh significantly activates Hedgehog signaling via the canonical Gli-1-dependent pathway in LEPCs. (**A–F**) LEPCs were cultured with 100 ng/ml Shh, and then canonical Hh target genes, including Gli-1, Ptc-1, VEGFA, and ANG1, and non-canonical Hh signal target genes, including OPN and MMP2, were analyzed using qRT-PCR. (**G–J**) HUVECs were cultured with 100 ng/ml Shh, and then the expression levels of Gli-1, OPN and MMP2 were analyzed. (**K**) LEPCs and HUVECs were cultured with 0–100 ng/ml Shh for 12 h, and then Gli-1 expression was assessed using Western blot analysis. (**L–N**) LEPCs and HUVECs were incubated with 200 ng/ml Shh and 20 ng/ml VEGF for 12 hours on matrigel and tube formation was observed under light microscope (scale bar, 100 μm).

It has been known that canonical Hh signaling is activated in eEPCs, while non-canonical signaling is activated in ECs treated with Hh ligands. However, the details of the Hh response in LEPCs have not been studied. Moreover, because it has been thought that LEPCs might merely be cells detached from vessel walls, molecular differences between LEPCs and HUVECs have not been thoroughly studied. We originally hypothesized that, as in HUVECs, non-canonical Hh signaling mediates the Ptc-1 activation in LEPCs. Because this has not previously been investigated, we examined the effect of Hh ligands on LEPCs. LEPCs were treated with Shh, and the expression of canonical and non-canonical Hh target genes was examined using qRT-PCR. Interestingly, we found that canonical Hh targets including Gli-1, VEGFA, Ang1, PTC-1 and HIP were up-regulated upon treatment with Shh, while OPN and MMP2, non-canonical target genes, showed no significant changes (Fig. 5). In contrast, as previously reported, HUVECs showed induction of the non-canonical Hh targets OPN and MMP2 upon Shh treatment without increased Gli-1 expression. Western blot results confirmed that Gli-1 expression was enhanced upon Shh treatment in LEPCs, while Shh treatment yielded no change in HUVECs (Fig. 5K). Together, these results suggest that LEPCs and ECs respond differently to Hh ligands and that canonical Hh signaling plays an important role in LEPCs. However, treating Shh increased tube formation of both LEPCs and HUVECs which indicates canonical and non-canonical pathway both leads activating angiogenesis (Fig. 5L).

### Overexpression of HIPin eEPCs Reduced Paracrine Effects on ECs

**Figure 6:**
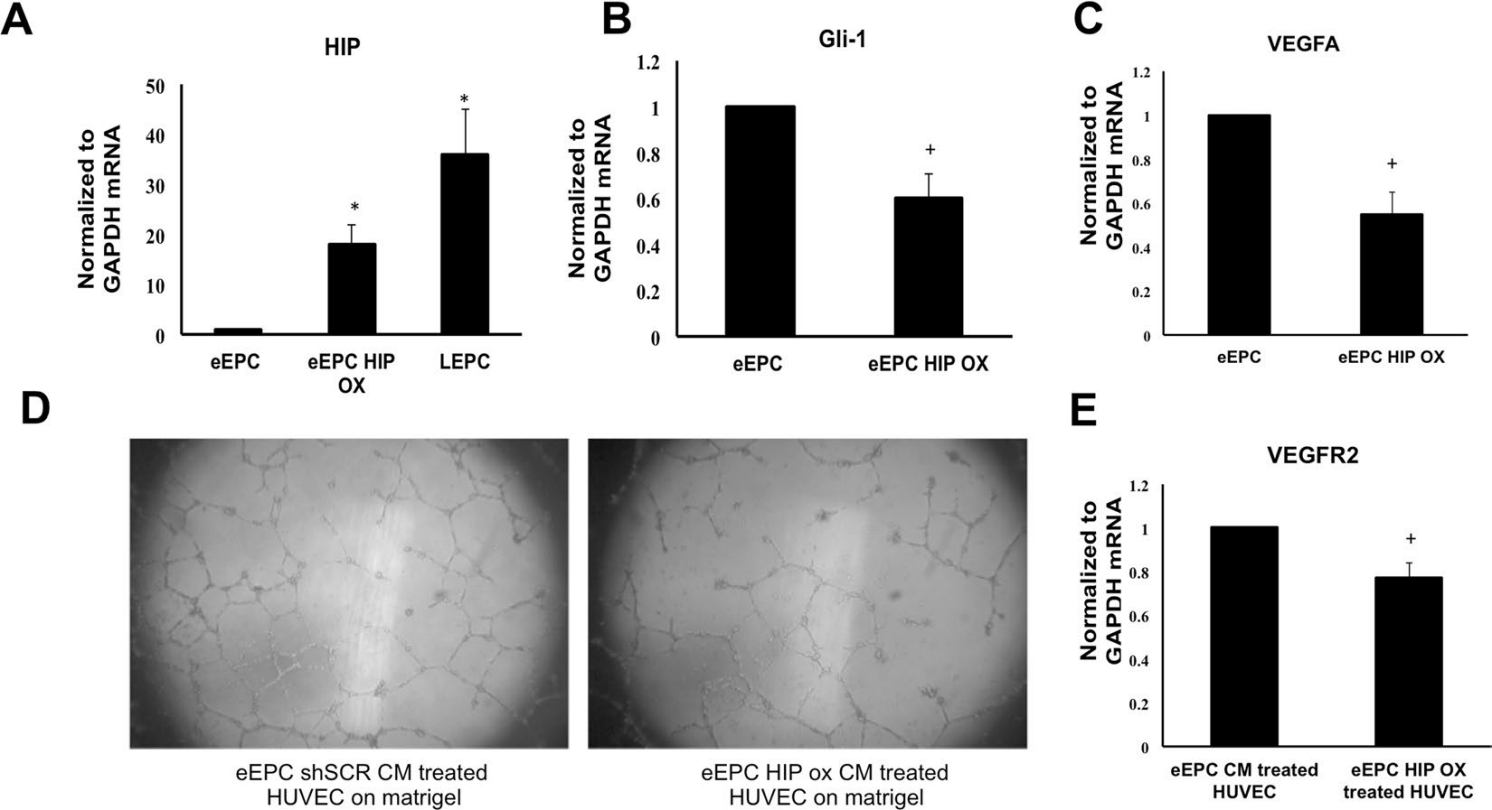
Overexpression of HIP in eEPCs impedes its paracrine angiogenic effect on ECs. The eEPCs were cultured for 6 days and then transfected with HIP overexpression vector for 24 h. After transfection, eEPCs were incubated in endothelial basal media without growth factors or with 2% FBS. (**A–C**) Expression levels of HIP, Gli-1 and VEGFA were measured using qRT-PCR. (**D**) Conditioned medium from eEPCs overexpressing HIP or from control eEPCs were applied to HUVECs suspended on Matrigel for 12 hrs in 96 well plate. Tube formation was visualized. (**E**) VEGFR2 expression in HUVECs treated with conditioned medium was assessed.

It has been reported that different roles of early and late EPCs contributed equally to neovasculogenesis *in vivo*. The eEPCs contribute to angiogenesis mainly by secreting cytokines that support the angiogenic function of resident mature ECs. Therefore, we tested the role of HIP in eEPCs in regards to angiogenesis. Because we demonstrated that HIP knockdown activates canonical Hh signaling and VEGFA expression in LEPCs, we tested the hypothesis that increased HIP expression in eEPCs reduces the expression of VEGFA and that release of VEGFA into the extracellular space would lead to subsequent induction of mature EC’s tube function. First, we treated eEPCs with a HIP-overexpression lentiviral vector and confirmed that HIP was overexpressed in eEPCs. HIP overexpression of more than 15-fold was detected, and subsequent low expression of Gli-1 was detected in eEPCs by qRT-PCR (Fig. 6A,B). Second, we verified the effect of eEPC-conditioned media on HUVECs; HUVECs treated with the conditioned media from eEPCs overexpressing HIP showed reduced tube formation (Fig. 6D). Third, we extracted RNA from the HUVECs treated for two days with the conditioned media ofeEPCs overexpressing HIP. Compared to the HUVECs treated with the conditioned media from normal eEPCs, HUVECs treated with media from eEPCs with high HIP expression showed reduced expression of VEGFR2, which is a direct downstream target of VEGFA signaling (Fig. 6E).

### HIP Knockdown Enhanced Angiogenesis and Apoptosis Evasion in LEPCs Through Activation of Canonical Hh Signaling

**Figure 7:**
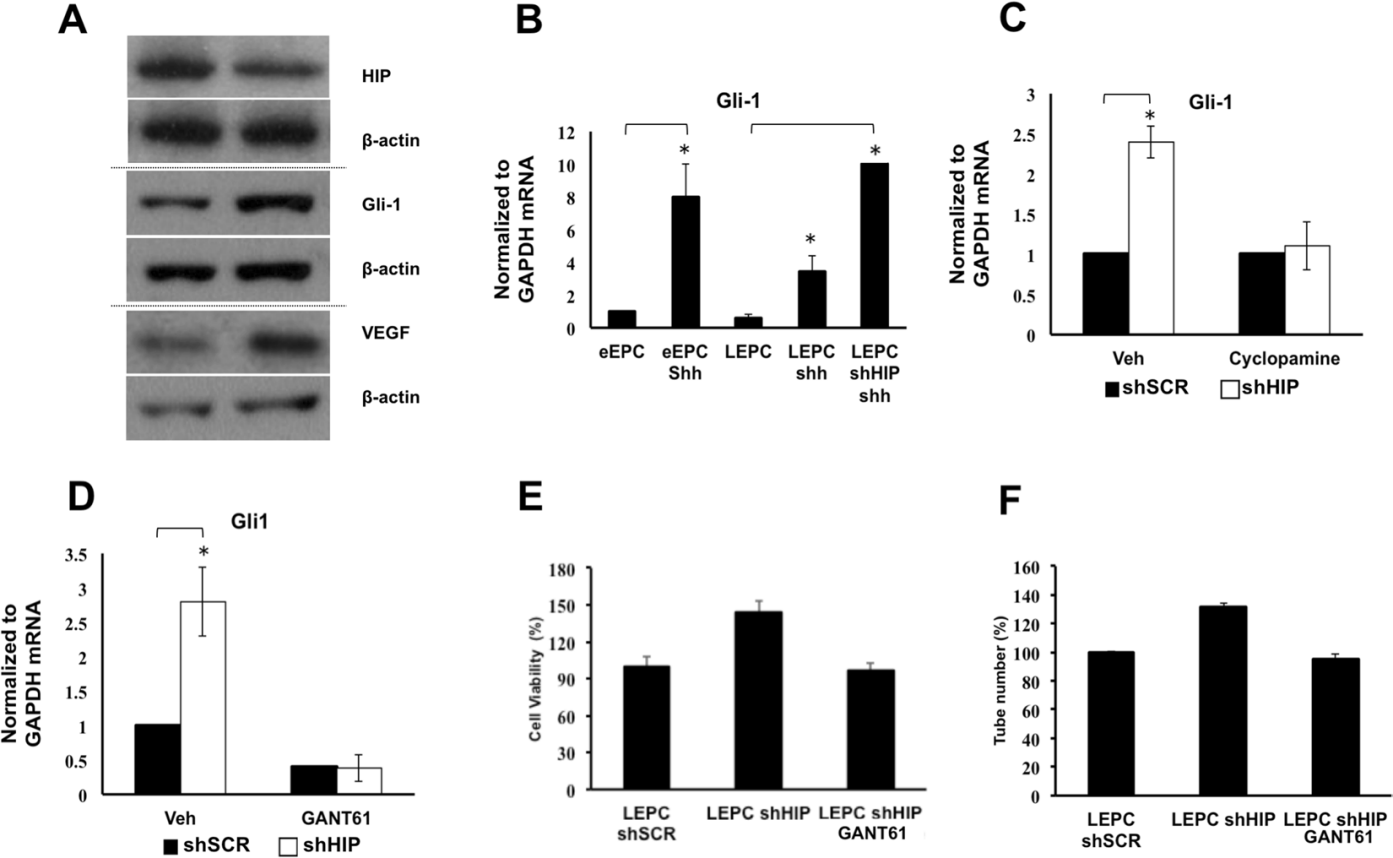
HIP knockdown activates canonical Hh target genes and enhances Hh responsiveness. (**A**) Inhibition of HIP increased the expression levels of Gli-1 and VEGFA. (**B**) eEPCs, LEPCs, and LEPCs with HIP knockdown were treated for 12 h with 100 ng/ml Shh, and then Gli-1 activation was assessed using qRT-PCR. (**C**,**D**) LEPCs and LEPCs with low HIP expression were treated with vehicle (DMSO), Hh inhibitor cyclopamine (20 µM) or Gli-1-specific inhibitor GANT61 (20 µM) for 12 h, and then Gli-1 expression was measured. (**E**) After treatment with 20 µM GANT61 for 24 hours, LEPCs expressing either scrambled or HIP shRNA were detached and seeded on Matrigel with 10 ng/ml VEGF. After 12 h, the tubes were counted. (**F**) After treatment with 20 µM GANT61 for 24 hours, LEPCs expressing either scrambled or HIP shRNA were treated with 50 µM H_2_O_2_ for 3 h, followed by the MTT assay.

Because we demonstrated that canonical Hh signaling is activated in LEPCs upon Shh stimulation, we hypothesized that HIP functions to block canonical Hh signaling in LEPCs. Indeed, we found that inhibition of HIP enhanced Gli-1 protein expression (Fig. 7A). Upon Shh treatment, Gli-1 mRNA was expressed at a high level in eEPCs, whereas it was low in LEPCs. This result suggests that high expression of HIP may inhibit Hh signaling in LEPCs given that their Ptc-1 expression levels were similar. Thus, when we treated LEPCs with Shh after knocking down the HIP, the Gli-1 expression was as high as in the eEPCs with Shh simulation, suggesting that inhibition of HIP enhanced the responsiveness of the Hh signal to Shh treatment (Fig. 7B). These results suggest that the high level of HIP in LEPCs, even with high expression ofPtc-1, efficiently blocks the canonical Hh signaling upon Shh stimulation. To further test the causal relationship between HIP and the expression of canonical Hh target genes, we used specific pharmacological Hh inhibitors, cyclopamine and GANT61. We found that the increase in Gli-1 expression induced by HIP knockdown was abolished either by the Smo inhibitor cyclopamine or the Gli inhibitor GANT61 (Fig. 7C,D). In addition, we found that the increase in angiogenesis and the decrease in apoptosis after oxidative stress induced by HIP knockdown were completely abolished by cyclopamine in LEPCs (Fig. 7E,F).

## Discussion

The characterization of EPC lineage has been most challenging and controversial. eEPCs that are isolated on fibronectin for 3-5 days highly expressed CD45 and CD14. However, as reported^19^ we observed lack of mature endothelial marker such as CD144 and CD146 (Supplementary Fig. 1A,B). It is supported by the recent finding that the endothelial gene promoters are silenced^20^. Although its active paracrine role of eEPC in vascular repair, its lack of endothelial marker suggest eEPC might not directly differentiate into endothelial cells. Therefore, in this study we focused analysis LEPCs during their differentiation. LEPCs were identified as colonies of endothelial cells appeared between 14 to 21 days of culture of umbilical cord derived MNCs.

In this study, we showed, for the first time to our knowledge, both RNA sequencing and proteome analysis LEPC lineage. Microarray data for LEPC samples have been reported in a previous study^21^. However, those data are limited to 8,880 genes and have no protein evidence. Comparative proteome analysis using LEPCs has been reported, but those data are limited to a total of 298 distinct spots on 2D-PAGE^22^. In the present study, based on the characterization of more than 23,000 genes and 2,127 proteins, we compared LEPCs to their precursor MNCs and to ECs. Interestingly, we observed that the gene expression profiles of these cell types committed to the endothelial lineage are very similar, suggesting the existence of a very subtle core gene expression profile that provide stage-specific distinctions (Fig. 1C). Functional characterization of endothelial lineage cells showed that MNCs are actively involved in the immune response, while genes related to cell adhesion and motility are activated in LEPCs and ECs (Fig. 1G and H). This is partly due to the nature of endothelial commitment and differentiation from the circulating blood MNCs because it requires coordinated multistep processes including mobilization, adhesion, transmigration and incorporation. By comparing LEPCs to eEPCs in FACs analysis ECs, we observed that LEPCs are more proliferative than ECs (Fig. 1I).

Among 101 specific markers of endothelial lineage that we confirmed based on both RNA and protein levels, we focused on HIP because of its relationship to Hh signaling. We found that HIP is strongly expressed in LEPCs and that inhibition of HIP increases LEPC tube formation and resistance to oxidative stress. Moreover, there has been no report of a specific molecular mechanism by which Hh signaling could contribute to LEPC function. In this study, we demonstrated that Shh activates the Gli-1 dependent canonical Hh pathway in LEPCs, whereas it activates only non-canonical Hh signaling in ECs^14^. To the best of our knowledge, this is the first report describing the differences in regulatory pathways between ECs and LEPCs. This difference might also explain why HUVECs are superior to LEPCs in terms of stable neovascularization^23^ and why LEPCs are superior in secreting angiogenic cytokines, given that non-canonical Hh signaling mostly modulates actin cytoskeleton-dependent processes such as migration and axonal extension, while Gli-dependent canonical Hh signaling is more involved in stem cell renewal, cell cycle regulation and cell survival.

Previous studies have indicated that HIP is highly expressed in adult heart, lung, brain, kidney and testis. HIP expression is decreased in several human tumors of the lung, stomach, colorectal tract, and liver compared with the corresponding normal tissues^11,13,19,21^. However, though HIP expression is high in LEPCs, its precise regulation mechanism and function in these cells has not been studied to date. In adult tissues, it has been reported that estradiol triggers Shh-induced angiogenesis during peripheral nerve regeneration by downregulating HIP^20^. HIP non-cell autonomously inhibits Hh-dependent neural progenitor patterning and proliferation^24^. These emerging lines of evidence suggest that HIP functions as an important inhibitor that temporally inhibit adult stem cell and progenitor cell function and differentiation. In this regard, we found that HIP is up-regulated during blood monocyte differentiation into the endothelial lineage. High expression of HIP in LEPCs negatively regulated LEPC angiogenesis. Interestingly, we found that HIP expression was decreased in LEPCs upon treatment with the angiogenic factors VEGF and FGF2 or when seeded on Matrigel. HIP expression was also decreased upon treatment with oxidative stress condition. The reduction of HIP expression by such angiogenic and stress triggers suggests that LEPC angiogenesis mediated by Hh signaling is tightly regulated and is initiated by angiogenic stimulus. A previous investigation indicated that VEGF and FGF2 are up-regulated in ischemic tissue and injury^25^. Oxidative stress can induce angiogenesis^26^.

Inhibition of HIP enhanced both tube formation and LEPC survival upon the induction of oxidative stress. As canonical Hh signaling positively regulates cell proliferation, HIP presumably regulates LEPC proliferation. Interestingly, without any stimulation, expression of HIP did not markedly affect LEPC proliferation (Supplementary Fig. 2). However, under oxidative stress, HIP affects LEPC survival and viability. Moreover, oxidative stress by itself decreases HIP expression. At the ischemic injury site, where circulating LEPCs are incorporated into the vascular structure and are actively involved in the repair process, ischemia-induced oxygen deprivation alters the defense mechanisms against oxygen free radicals. Simultaneously, production of oxygen free radicals increases vascular and tissue damage. We found that when using H_2_O_2_ treatment to mimic such emergency conditions, inhibition of HIP enhanced LEPC survival and viability.

The vascular endothelium lines the entire circulatory system, and endothelial cells turn over very slowly to maintain homeostasis. Rapid proliferation and angiogenesis are initiated only after ischemic disease or injury. In this regard, it is suggested that HIP functions to maintain LEPCs at steady state. Only with a proper angiogenic trigger, such as VEGF and FGF2 or seeding on Matrigel, HIP expression was reduced, thereby greatly affecting LEPC angiogenesis. Under physiological conditions, HIP does not affect LEPC viability and proliferation, but under oxidative stress, HIP knockdown markedly affects LEPC viability and survival.

In conclusion, using a combined transcriptomic and proteomic approach, we identified a 101-gene endothelial signature that could be further used to characterize endothelial commitment. Among these genes, our data first show that HIP is a strong negative regulator of LEPCs through regulation of Gli-dependent canonical Hh signaling. On the one hand, HIP knockdown in LEPCs improves LEPC angiogenesis. On the other hand, HIP knockdown enhances LEPC survival under oxidative stress. Strong expression of HIP is down-regulated upon angiogenic stimulus. This study provides mechanistic insight into the regulation of LEPC function and identifies novel markers that may have potential as therapeutic targets.

## Materials and Methods

### Cell culture

Mononuclear Cells (MNCs), Late endothelial progenitor cells (LEPCs) and early endothelial progenitor cells (eEPCs) were isolated from human umbilical cord blood as previously described^27^. Fresh human cord blood was obtained from pregnant female volunteers, without significant disease, not receiving any medication and without any clinical diagnosis. All patients gave informed consent. All the procedure and protocol was approved by Daegu Fatima Hospital and Seoul National University (IRB No. E1403/001-010). Human umbilical vein endothelial cells (HUVECs), LEPCs and MNCs for the initial screening were provided by Dr. SM Kwon at the College of Medicine, Pusan National University. MNCs were resuspended in Endothelial cell growth medium (EGM™-2 MV not including hydrocortisone, Lonza) supplemented with 2% fetal bovine serum (FBS) onto 6-well tissue culture plate pre-coated with fibronectin (BD Bioscience) at 37 °C, 5% CO_2_, in a humidified incubator. After 24 hours of culture, non-adherent cells were aspirated and medium was changed daily. Adherent cells incubated for 3 to 5 days were used as eEPCs. Colonies of endothelial cells appeared between 14 to 21 days of culture and were identified as LEPCs. LEPCs and HUVECs were maintained on dishes coated with 1% gelatin (Sigma). EGM™-2 medium was changed every 48 hours.

### RNA-sequencing analysis

Total RNA was extracted from MNCs, MACS-sorted CD146 + cells LEPCs and HUVECs using TRIzol reagent (Life Technologies). The total RNA was treated with DNase I and then was purified with a miRNeasy Mini Kit (Qiagen). The quality of the RNA was checked using an Agilent 2100 Bioanalyzer (Agilent) prior to sequencing on the Illumina platform for transcriptomics with a 90-bp paired-end library (Illumina). Libraries were constructed following the Illumina Paired-End Sequencing Library Preparation Protocol. Library quality and concentration were determined using an Agilent 2100 BioAnalyzer. Each sample was sequenced via paired-end sequencing with an Illumina HiSeq. 2000 using HiSeq Sequencing kits.

### Proteome analysis

Proteins were extracted from MNCs, MACS-sorted LEPCs, and HUVECs using RIPA buffer (Thermo Fisher) according to the manufacturer’s instruction. Quick Start™ Bradford 1x Dye Reagent (Bio-Rad Laboratories) was used to measure protein concentration. Then, 120 µg of proteins was loaded, in SDS PAGE gel and 30 fractions (SDS-PAGE) were further analyzed on an LTQ-Orbitrap (Thermo Fisher) as previously described^28^. The datasets generated by the LTQ-Orbitrap were analyzed using Scaffold (version 4.4.1, Proteome Software, Inc.) Peptide identifications were accepted with 90.0% probability and FDR less than 1.0% by a Scaffold local FDR algorithm with at least 2 identified unique peptides.

### shRNA transfection and construction of the HIP overexpression vector

Human LEPCs were transfected with a specific HIP shRNA (sc-43835-SH) or with control shRNA (sc-108060, used as transfection control) for 24 hours in EGM2 media. LEPCs were incubated for 48 h after the transfection, and Puromycin (50 ng/ml) was added for selecting transfected LEPCs. To create the Hedgehog-interacting protein (HIP) expression vector, HIP DNA was purchased from the Korea Human Gene Bank (KRIBB). Human HIP was amplified using the forward primer 5′ CGACTAGTTCTAGAATGCTGAAGATGCTCTCCTTTA and the reverse primer 5′-GAGGGGCGGGATCCCTATACAATGTAACTTGTTAC. The amplified Hipgene was inserted into pcDNA3.1.eEPCs were transfected at day 6 of cell culture with pcDNA3.1 encoding the full Hip gene or with pcDNA3.1 without Hip for 24 hours in EGM2 media.

### RT-PCR and quantitative RT-PCR Analysis

Total RNA was extracted using Trizol reagent (Invitrogen) according to the manufacturer’s instructions. For each sample, 1 µg of total RNA was used for cDNA synthesis with random hexamers using the Omniscript RT kit (Qiagen). PCR was performed in a thermocycler (Bio-Rad) with GoTaq® DNA polymerase (Promega). The relative expression of each mRNA was calculated by the comparative threshold cycle (CT) method. One-way ANOVA was performed, and gene lists were created using a P value with a false discovery rate < 0.01.

### Tube-formation assay

Tube-formation assay on Matrigel was performed as previously reported^25^. In brief, culture plates were coated with 70 μl of growth factor-reduced Matrigel™ (BD Biosciences) per well. LEPCs were seeded on Matrigel-coated plates at a density of 2.5 × 10^4^ cells per well in EGM-2 plus 2% FBS in the presence of 20 ng/ml VEGF (R&D), followed by incubation at 37 °C for 24 h. Tube formation was examined by light microscopy 12 h later.

### Aortic sprouting assay

Mouse thoracic aortas were dissected from 6- to 8-week-old male C57BL/6 mice as previously reported. The aortas were immediately transferred to Petri dishes, and the adventitia and small vessels around the aorta were carefully removed. Aortas were incubated in EGM2 containing lentivirus and polybrene (8 µg/ml) for overnight. The resulting virus-transduced aortic rings were embedded in 150 μl of Matrigel in EGM2 media with 20 ng/ml VEGF in 96-well plates. The plates were incubated at 37 °C in a humidified 10% CO2 atmosphere. Medium containing VEGF was replaced 3 times per week. After 7 days, micrographs of representative rings were taken, and the total numbers of vascular sprouts and branch points were calculated. All mouse experiments were performed in accordance with the international guidelines and regulations for the use of experimental animals, and the protocol was approved by institutional animal care and use committee of Seoul national university

### Migration assay

Boyden chamber migration of LEPCs was performed as  previously described^29^. In brief, LEPCs per well in 300 µl of medium were added to the top chambers of 24-well transwell plates (5.0 µm pore size; Costar). EGM-2 plus 2% FBS and 20 ng/ml of VEGF was added to the lower chambers. After 24 hours of incubation at 37 °C with 5% CO_2_, the lower chambers were imaged.

### Western Blotting

Western blotting was performed as previously described^30^. Cells were lysed in RIPA buffer (Thermo Fisher) with protease inhibitor cocktail (Roche). The supernatant of the lysate was collected and denatured with the same volume of SDS sample buffer (0.5 M Tris-HCl, pH 6.8; 10% SDS, 50% glycerin, 2-mercaptoethanol). The protein samples were resolved viaSDS-PAGE and blotted onto PVDF membranes (Bio-Rad). PVDF membrane was cut to enable blotting for multiple antibodies simultaneously which were then labeled with the primary antibody overnight at 4 °C. Anti-HIP antibody (Abcam, Ab39208), Anti-Gli1 antibody (Biolegend, 642401), Anti-VEGF antibody (Biolegend, 512901), and Anti-Caspase-3 antibody (Abcam, Ab90437) at a dilution of 1:1000 were used. Then secondary antibody was incubated for 1 h at RT and detected using ECL Plus reagents(Thermo Fisher).

### Cell viability assay after oxidative stress

LEPCs were treated with H_2_O_2_ (100 mM) for 3 h. Then, cell morphology was observed under a light microscope. Cell viability was measured via the MTT assay (Sigma) to determine relative cell growth after 24 hrs. Then, 100 μl of 0.2 mg/ml MTT was added to the media for 5 h of incubation at 37°. After removal of the culture medium, the remaining crystals were dissolved in 500 μl of DMSO (Duksan), and absorbance at 470 nm was measured.

### Flow Cytometry analysis

Cells were treated with 0.05% trypsin-EDTA (Invitrogen) for 3 minutes in a 37 °C incubator. Cells were dissociated by gentle pipetting and filtered through a 40-μm cell strainer. The dissociated cells were immediately resuspended at approximately 2 × 10^5^ cells per ml in PBA (1% bovine serum albumin, 0.02% NaN_3_ in PBS) and incubated with each MAbs (anti-human CD14-FITC (BD Biosciences, Cat. No. 557153), anti-human CD31-FITC (BD Biosciences, Cat. No. 560984), anti-human CD105 (BD Biosciences, Cat. No. 561443), anti-human CD117-PE (BD Bioscience, Cat. No. 340529), anti-human CD146-FITC (BD Bioscience, Cat. No. 560846), anti-human VEGFR2-FITC (Miltenyi Biotec, Cat. No. 130-105-303), anti-human CD144-FITC (BD Bioscience, Cat. No. 560874), anti-Human CD34-FITC (BD Bioscience, Cat. No. 555821), anti-human CD45-FITC (Biolegend, Cat. No. 368508) for 30 minutes at 4 °C. After washing twice with PBA, propidium iodide (PI)–negative cells were analyzed for the antibody binding using FACSCalibur (BD Biosciences – Immunocytometry Systems, and Cell Quest software (BD Biosciences - immunocytometry Systems).

### Statistical analyses

Data are presented as the means ± SD, and statistical comparisons between groups were performed using one-way ANOVA. *Indicates p < 0.05 vs. the control group, and +indicates p < 0.05 vs. the control group in one-way ANOVA.

## Electronic supplementary material

Supplementary Figure Information

Supplementary Table 1
