# Supplementary material for: Hedgehog-Interacting Protein (HIP) Regulates Apoptosis Evasion and Angiogenic Function of Late Endothelial Progenitor Cells
Source: Sci Rep. 2017 Sep 29;7:12449. doi: 10.1038/s41598-017-12571-5 (PMC5622095; doi:10.1038/s41598-017-12571-5)
Supplement: Supplementary file 1 — Supplementary Figure Information [file 41598_2017_12571_MOESM1_ESM.doc]

Hedgehog-Interacting Protein(HIP) Regulates Apoptosis Evasion and Angiogenic Function of Late Endothelial Progenitor Cells.

Bom Nae Rin Lee^1#^, Yeon Sung Son^1#^, Dabin Lee^1^, Young-Jin Choi^1^, Sang-Mo Kwon^2^ , Hyun-Kyung Chang, Pyung-Hwan Kim and Je-Yoel Cho^1†^

**Supplementary Figure S1** Characterization of eEPC and LEPC by flow cytometry. FACS analysis of cell surface marker CD14, CD31, CD117, CD105, CD146, CD45, CD34, CD144, VEGFR2/KDR


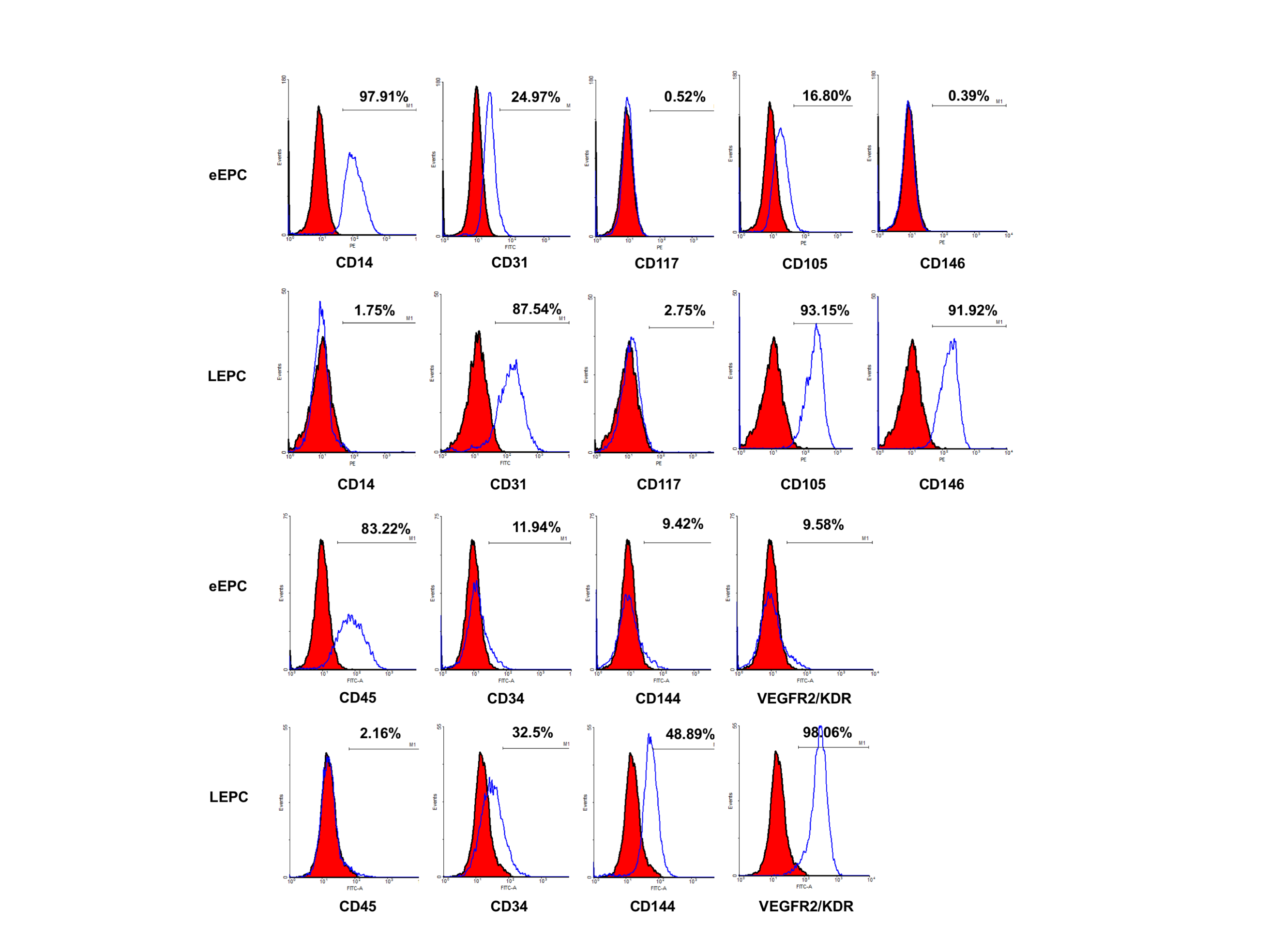


**Supplementary Figure S2. Inhibition of HIP does not affect LEPC migration orproliferation.**

**(A)**The migration of LEPCs with HIP knockdownwas assessed using thescratch (wound healing) assay.**(B)**The migration of LEPCs with HIP knockdownwas visualized using thetrans-well migration assay.**(C)**The proliferation of LEPCs with HIP knockdownwas assessed using theMTT assay. **(D)-(E)**Expression of Cyclin D and Cyclin E after HIP knockdownwas measured using qRT-PCR.


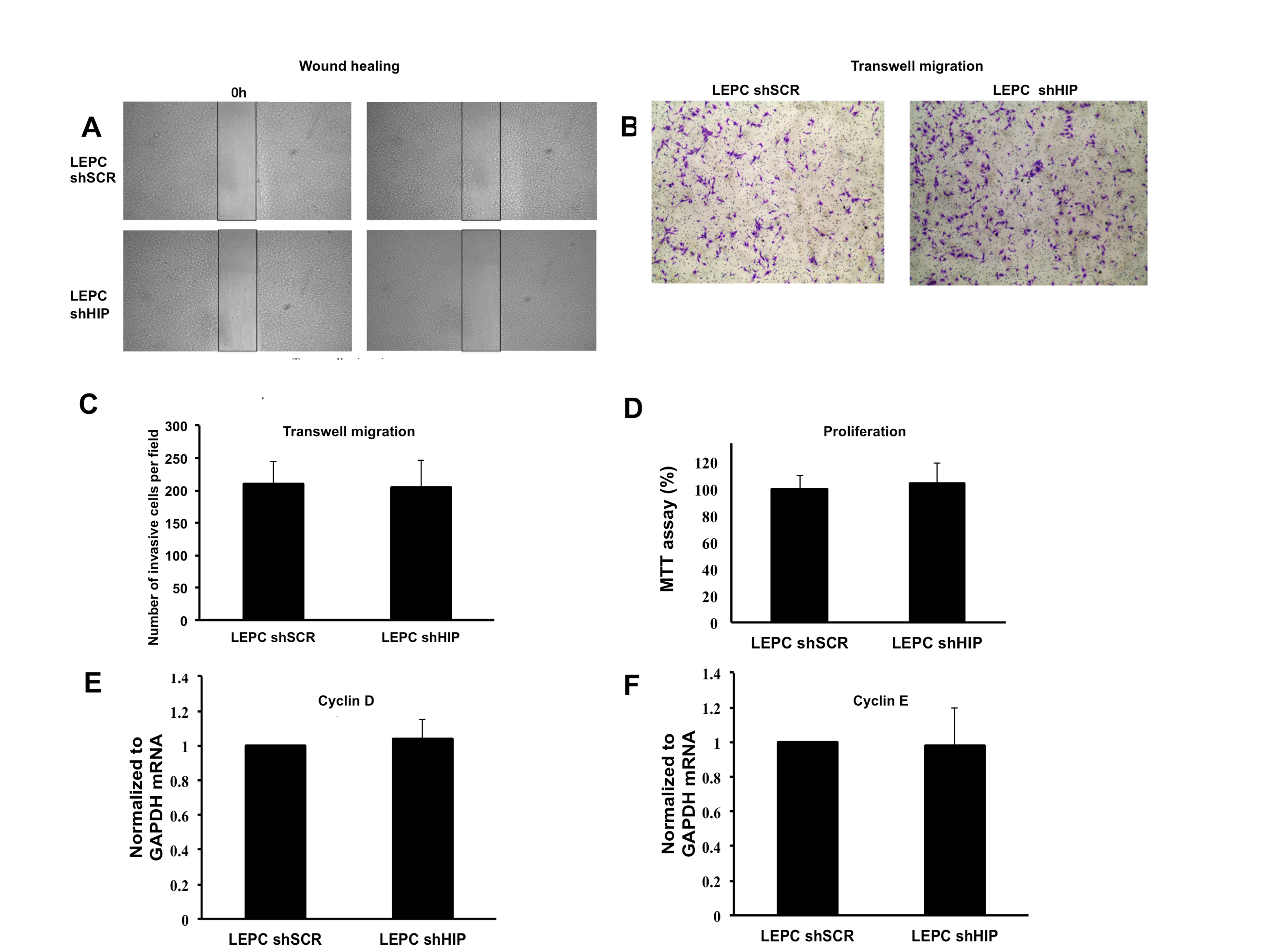


**Supplementary table 1**

To identify stage-selective LEPC markers and endothelial markers, we compared genes showing 2-fold upregulation in both the transcriptome (FPKM) and proteome data (spectrum count) sets. We found 244 genes that were up-regulated in LEPCs compared to MNCs in both RNA and protein levels. Next, we compared HUVECs and MNCs. We found 164 genes that were up-regulated in HUVECs compared to MNCs in both the transcriptome and proteome data sets. Finally,among those 244 genes, We identified 101 genes as common to LEPCs and HUVECs.
